# Reply to Guidotti, T. Comment on “Laroche, E.; L’Espérance, S. Cancer Incidence and Mortality among Firefighters: An Overview of Epidemiologic Systematic Reviews. *Int. J. Environ. Res. Public Health* 2021, *18*, 2519”

**DOI:** 10.3390/ijerph19137560

**Published:** 2022-06-21

**Authors:** Elena Laroche, Sylvain L’Espérance

**Affiliations:** 1 Department of Management, Université Laval, Québec, QC G1V 0A6, Canada; 2 CHU de Québec, Université Laval, Québec, QC G1L3L5, Canada; syles@hotmail.com

We have reviewed the comment [1] written to the Editor in reference to our original article entitled: “Cancer Incidence and Mortality among Firefighters: An Overview of Epidemiologic Systematic Reviews” [2] and thank you for the opportunity to respond to the comments.

The objective of the article is “to assess the conclusion consistency across the available systematic reviews on the cancer risk in firefighters”. Since several original studies assessed the risk of cancer incidence or mortality in firefighters and many systematic reviews (SRs) have been produced with sometimes conflicting conclusions, this specific objective may be relevant to identify gaps and better guide future research. The methodology used was a systematic review method, conducted in accordance with the Preferred Reporting Items for Systematic Reviews and Meta-Analyses (PRISMA) [3]. The results highlighted the importance of having a rigorous methodology for conducting systematic reviews. However, because it was a review of systematic reviews, it did not identify gaps in the original 104 studies, focusing primarily on the 11 systematic reviews listed in our overview.

The commentary specifies that “chemical exposures, firefighting practices, and health protection have changed dramatically in recent years” and that this was not considered in the article. We agree with this, as we noted in the discussion section, where we stated that “improvements in fire control practices and changes in the worker’s personal protective equipment could potentially have had an impact on the observed results. Exposure to the various carcinogens present during a fire may also have changed over time”.

Dr. Guidotti’s systematic review [4] included in our work is certainly a detailed and valuable report on the issue. At the outset of the report, it is stated that the primary objective of the report is “to conduct a systematic review of the world literature on firefighting”. As this work met our inclusion criteria, the report was included in the systematic review. Dr. Guidotti’s report also provides a relevant and more advanced analysis of the field. Although our results identified some methodological concerns (assessed using the ROBIS tool [5]), we understand that the report was prepared for a government and noted that a descriptive study such as Dr. Guidotti’s is very relevant to this type of research. On the other hand, we are sorry if Dr. Guidotti’s other work [6,7] did not meet our inclusion criteria (see Appendix D of the original study) or literature search strategies (see Appendix C of the original study), as they do not include a systematic literature search methodology. This in no way diminishes the relevance and value of this work. 

As mentioned, the objective of the original article was to assess the conclusion consistency across the available systematic reviews on the cancer risk in firefighters. To our knowledge, this had not been accomplished before and, in this respect, constitutes an advancement to knowledge in the field. Our review does not go into depth in the study of the various types of cancer in firefighters, but the method chosen is consistent with the objectives pursued. The objective was to review systematic reviews on the subject and we assessed the quality of the studies included (using ROBIS). 
